# Society Meetings

**Published:** 1900-04

**Authors:** 


### SOCIETY MEETINGS.

The American Academy of Medicine will hold its twenty-fifth annual meeting at "The Shelburne," Atlantic City, N.J., on Saturday, June 2d and Monday, June 4, 1900.

Papers have been promised as follows: President's address; Contributions to the Annual Symposium—The medical aspects of the home, G. Hudson Makuen, Philadelphia; What are the essential conditions for a habitation to develop and maintain a healthful family existence? Rosa Engelmann, Chicago; The influence of early training of manly and womanly qualities to avoid degeneracy," J. Cheston Morris, Philadelphia; Artificial lighting of the home, S. D. Risley, Philadephia; The influence of medical supervision of children in their homes, J. Madison Taylor, Philadelphia; The physician's influence *in re* vacation schools, Helen C. Putnam, Providence; Defectives and delinquents inside and outside the family circle, James W. Walk, Philadelphia.

*Papers on Miscellaneous Topics.*—Is coöperation between colleges of arts and sciences and schools of medicine desirable? A. L. Benedict, Buffalo; Physiologic psychology as a necessary element in medicine, W. J. Herdman, Ann Arbor; Medical education of today, Bayard Holmes, Chicago; Neglected clinical opportunities in American medical centers, S. A. Knopf, New York; Subject to be announced. W. L. Pyle, Philadelphia; The physician vs. medical proprietors and medical patentees, A. Ravogli, Cincinnati; Medicine in the Philippines, Harry Park Ritchie, St. Paul, recently on medical duty with the U. S. V. in Luzon; Report of the special committee to formulate the conclusion reached regarding specialism and advertising at the last meeting of the Academy.

There is room for a few additional papers without crowding the program. Some of these are promised provisionally, but are not included in the list. Anyone desiring to contribute a paper should at once notify the secretary, Dr. Charles McIntire, Easton, Pa., as the policy of the Academy is not to overcrowd the program.

---

THE Buffalo Academy of Medicine held meetings during the month of March, 1900, as follows:

*Section on Surgery.*—Tuesday evening, March 6th. Program: Spina bifida, Sidney A. Dunham.

*Section on Ophthalmology, Otology and Laryngology.*—Monday evening, March 12th. Program: Outdoor relief for eye and ear cases, Lucien Howe; Exhibition of cases—(1) Chronic suppuration of middle-ear, treated by Stacke-mastoid operation three weeks since; (2) Chonic empyema of nasal accessory sinuses, in which antrum, frontal sinus, ethmoid cells, and sphenoidal sinus have been opened, F. W. Hinkel.

*Section on Medicine.*—Tuesday evening, March 13th. Program: Senile bronchitis, Reynold W. Wilcox, professor of medicine and therapeutics, New York Post-Graduate Medical School; discussion by Charles Cary, Charles G. Stockton and Henry C. Buswell.

*Section on Pathology.*—Tuesday evening, March 20th. Program: The present status of recent investigations on cancer, with demonstrations by stereopticon, H. R. Gaylord.

*Section on Obstetrics.*—Tuesday evening, March 27th. Program: The early days of ovariotomy; reminiscences of McDowell, Peasley and others, Matthew D. Mann. Subject to be announced. Helene Kuhlmann.

The Niagara Falls Academy of Medicine held its regular meeting at the office of Dr. F. R. McBrien, 113 Falls Street, Monday, March 5th, at 8.45 p.m. Dr. McBlain read a paper on Ptomaines, and the discussion was opened by Dr. Leo-Wolf.

---

The Congress against Tuberculosis will meet at Naples on the 25-28 of April, 1900, under the presidency of his Excellency Minister Baccelli. The general committee, presided over by Senator Prof. de Renzi, and of which Prof. A. Rubino is the secretary, superintends the arrangements of the congress, the aim of whose labors is the same as that first set on foot at the congress of Berlin. It will be divided into the following sections, each of which has a separate committee of management: etiology and prophylaxy; clinical pathology; therapeutics; sanatories.

In this congress are entitled to take part: physicians, naturalists, engineers, as well as representatives of the social and philanthropic sciences. The subscription for each person is 20 francs (Italian) and entitles to the member's ticket and badge of the congress, as well as to the reduced fares on the Italian railways and steamboats, to the transactions of the congress, and all publications connected with the same, as well as the free entrance to the museums, Pompeii, Herculanum, etc., etc.

Applications as well as the subscriptions are to be sent to the secretary's office of the general committee, I. Clinica medica della R. Università di Napoli (*Ospedale Clinico.*)

